# DNA methylation markers panel can improve prediction of response to neoadjuvant chemotherapy in luminal B breast cancer

**DOI:** 10.1038/s41598-020-66197-1

**Published:** 2020-06-08

**Authors:** Vladimir O. Sigin, Alexey I. Kalinkin, Ekaterina B. Kuznetsova, Olga A. Simonova, Galina G. Chesnokova, Nikolai V. Litviakov, Elena M. Slonimskaya, Matvey M. Tsyganov, Marina K. Ibragimova, Ilya V. Volodin, Ilya I. Vinogradov, Maksim I. Vinogradov, Igor Y. Vinogradov, Sergey I. Kutsev, Vladimir V. Strelnikov, Dmitry V. Zaletaev, Alexander S. Tanas

**Affiliations:** 1 Research Centre for Medical Genetics, 115522, Moskvorechie St.1, Moscow, Russian Federation; 2 I.M. Sechenov First Moscow State Medical University, 119991, Trubetskaya St.8, Moscow, Russian Federation; 3 Tomsk Cancer Research Institute, 634009, Kooperativniy Lane, 5, Tomsk, Russian Federation; 4 National Research Tomsk State University, 634050, Lenin Ave, 36, Tomsk, Russian Federation; 5 Pathology and Anatomy Department with Pathology Laboratory, Ryazan Regional Clinical Oncology Dispensary, Sportivnaya St.13, Ryazan, 390011 Russian Federation; 6 Ryazan State Medical University, Vysokovoltnaya St.9, Ryazan, 390026 Russian Federation

**Keywords:** Breast cancer, Epigenomics, DNA methylation

## Abstract

Despite the advantages of neoadjuvant chemotherapy (NACT), associated toxicity is a serious complication that renders monitoring of the patients’ response to NACT highly important. Thus, prediction of tumor response to treatment is imperative to avoid exposure of potential non-responders to deleterious complications. We have performed genome-wide analysis of DNA methylation by XmaI-RRBS and selected CpG dinucleotides differential methylation of which discriminates luminal B breast cancer samples with different sensitivity to NACT. With this data, we have developed multiplex methylation sensitive restriction enzyme PCR (MSRE-PCR) protocol for determining the methylation status of 10 genes (*SLC9A3, C1QL2, DPYS, IRF4, ADCY8, KCNQ2, TERT, SYNDIG1, SKOR2* and *GRIK1*) that distinguish BC samples with different NACT response. Analysis of these 10 markers by MSRE-PCR in biopsy samples allowed us to reveal three top informative combinations of markers, (1) *IRF4* and *C1QL2*; (2) *IRF4, C1QL2*, and *ADCY8*; (3) *IRF4, C1QL2*, and *DPYS*, with the areas under ROC curves (AUCs) of 0.75, 0.78 and 0.74, respectively. A classifier based on *IRF4* and *C1QL2* better meets the diagnostic panel simplicity requirements, as it consists of only two markers. Diagnostic accuracy of the panel of these two markers is 0.75, with the sensitivity of 75% and specificity of 75%.

## Introduction

Preoperative (neoadjuvant) chemotherapy (NACT) is recommended for patients with locally advanced breast cancer (BC) of IIIA, IIIB, or IIIC stages and can be recommended for patients with IIA (T2N0 or T1N1) or IIB (T2N1 or T3N0) stages with triple-negative, luminal B, HER2-positive BC, and in cases with lymphogenous metastases^1,2^. NACT is capable of providing reduction of the tumor volume, which is one of the key missions of NACT in terms of downstream conservation surgery, and of reducing distant metastases. Patients whose tumors completely disappear after NACT, demonstrate longer disease-free survival than those with residual tumors^3,4^. Despite the advantages of neoadjuvant chemotherapy (NACT), associated toxicity is a serious complication that renders monitoring of the patients’ response to NACT highly important. Thus, prediction of tumor response to treatment is imperative to avoid exposure of potential non-responders to deleterious complications. The overall response to chemotherapy varies from 69–100%, according to the results of clinical trials with different treatment regimens. Yet, complete pathological response rates ranged from 10% to 31%^5,6^. This highlights the need for early prediction of treatment response and importance of identification of molecular markers that would predict the sensitivity of the tumor to neoadjuvant chemotherapy by analysis of biopsy material obtained prior to treatment.

The studies of predictive factors for the NACT effectiveness, which are complementary to the conventional clinical and pathological factors, are focused on the phenomenon of multidrug resistance (MDR). Assays to predict tumor response to NACT have been developed, which are based on the detection of MDR genes deletions and down-regulation of their expression^7^.

As an additional source of predictive markers, we consider methylotyping of malignant tumors. DNA methylation profiles of tumor genomes have already been used in epigenetic classifications of BC subtypes^8,9^.

Effective and truly unbiased selection of the DNA methylation markers that would discriminate the cohorts of clinical samples under comparison is only possible with the genome-wide differential methylation screening assays, preferably based on DNA sequencing, yet whole-genome bisulfite sequencing is not likely to be the method of choice in methylation biomarkers development. Reduced representation bisulfite sequencing (RRBS) increases the relative information value of DNA methylation analysis by NGS, as compared with the whole-genome bisulfite sequencing: every RRBS sequence read includes at least one informative CpG position^10^. In contrast to the whole genome approach, RRBS libraries are generated using a specific restriction endonuclease MspI that forms a pool of the CpG-rich DNA fragments. This significantly decreases the fraction of a genome undergoing sequencing and enriches it with the most relevant regions (CpG islands). Theoretically, RRBS is better than whole-genome bisulfite sequencing, applicable to the large-scale studies of DNA methylation, a marker of epigenetic processes in health and disease, as far as RRBS focuses on the CpG islands that constitute a minor fraction of the genome and omits its less clinically relevant major part^10^.

In 2015, we have developed a method of reducing the size of the RRBS library without a significant loss of the CpG islands. We designated the method “XmaI-RRBS” because it involves the XmaI restriction endonuclease for library preparation in contrast to the classical approach that involves the MspI endonuclease. Sequencing of the XmaI-RRBS library of the fragments with optimal length of 110-200 base pairs results in simultaneous calling of the methylation status of over 125000 CpG dinucleotides, over 90000 belonging to CpG islands^10,11^. XmaI-RRBS allows rapid and affordable genome-wide bisulfite DNA sequencing for assessing the methylation of human CpG islands in significant collections of clinical samples^10^. In the present study, we adopted the XmaI-RRBS method to perform a genome-wide search for DNA methylation markers that distinguish biopsy specimens of BC obtained prior to treatment by the effectiveness of subsequent NACT.

Despite the utility of the RRBS method in terms of developing epigenetic classifiers of malignant tumors, the feasibility of genome-wide analysis of DNA methylation in the clinical setting is vague. In this regard, the ultimate aim of this study was to develop a simple and clinically applicable test for predicting the effectiveness of neoadjuvant chemotherapy for BC based on a limited set of DNA methylation markers assessed by the method of multiplex methylation sensitive restriction enzyme PCR (MSRE-PCR).

## Results

**Figure 1:**
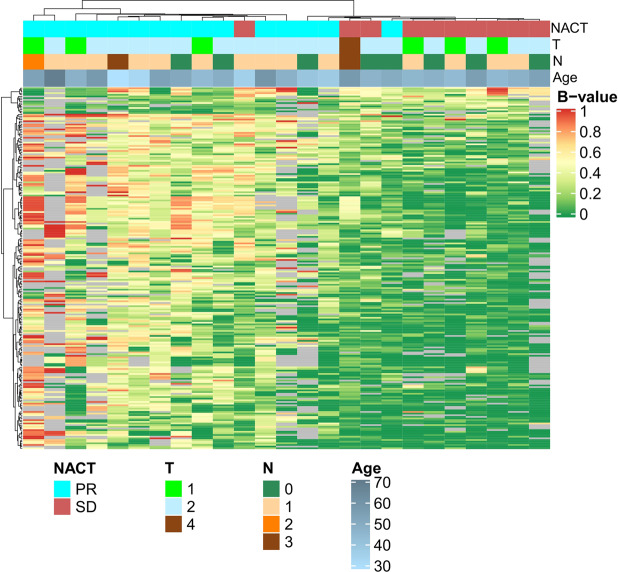
The heat map of methylation levels (b-values) of CpG dinucleotides in luminal B breast cancer samples with different response to neoadjuvant chemotherapy (NACT) identified in this study using XmaI-RRBS method. The red bars and green bars represent hypermethylation CpG sites and hypomethylation CpG sites, respectively. Data are presented in a matrix format: each row represents a CpG dinucleotide differential methylation of which marks the differences in the responses of BC tumors to NACT, and each column, a tumor sample. Patients’ age, tumor size (T) and lymph node status (N), response to NACT are indicated as colored rectangles.

On 25 samples (Cohort 1) of luminal B breast cancer biopsies taken before treatment, a genome-wide analysis of DNA methylation using the XmaI-RRBS method^10,11^ was performed. Based on the obtained methylation data for over 100,000 CpG dinucleotides, genome positions were selected, the differential methylation of which is observed in groups of BC samples with different sensitivity to NACT (Fig. 1). Selected CpG dinucleotides belong to promoter regions of 15 genes, *CNIH3, PRR5, PTGIS, ATOH1, SNAP25, SLC9A3, C1QL2, DPYS, IRF4, ADCY8, KCNQ2, TERT, SYNDIG1, SKOR2* and *GRIK1*.

**Figure 2:**
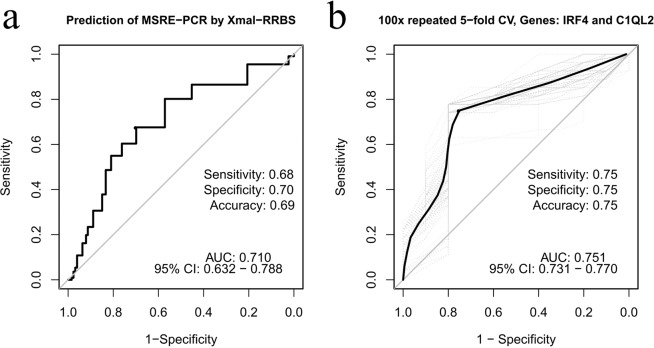
ROC curve for the XmaI-RRBS and MSRE-PCR methods comparison (**a**) and evaluation of a two-gene (*IRF4* and *C1QL2*) system for the prediction of luminal B breast cancer response to NACT by ROC analysis with 100x repeated 5−fold cross-validation (**b**).

In order to provide a clinically applicable tool to determine the methylation status of gene loci selected by XmaI-RRBS as the most informative markers of BC NACT sensitivity, we have developed a test system based on a multi-locus MSRE-PCR targeting the 15 genes listed above. Assessment by this MSRE-PCR assay of the same 25 BC samples that at the previous step underwent XmaI-RRBS analysis resulted in positive methylation calling from *PRR5, PTGIS, ATOH1, CNIH3* and negative methylation calling from *SNAP25* in all samples, an artifact that may be expected with the transition to another technology. Exclusion of these loci from the assay would not impact the quality of the classifier, yet we have not excluded them from the MSRE-PCR assay as they provide additional technical PCR amplification and DNA digestive controls. Assessment of the remaining 10 markers with MSRE-PCR demonstrated acceptable reproducibility of the results compared to the XmaI-RRBS (AUC = 0.710 with sensitivity, specificity, and accuracy of 68%, 70% and 69% respectively; Fig. 2a).

DNA methylation data obtained by MSRE-PCR for 10 markers in a Cohort 2 of 37 luminal B breast cancer biopsy samples taken before treatment for all of which NACT response was later reported were used for the development of a classifier and for assessment of its diagnostic utility by cross-validation. The methylation frequencies of 10 informative genes (SLC9A3, C1QL2, DPYS, IRF4, ADCY8, KCNQ2, TERT, SYNDIG1, SKOR2 and GRIK1) determined in a set of 37 luminal B breast cancer samples with different NACT response shown in OFig. 3 suggest unequal potential of individual markers in discrimination of NACT responding and non-responding tumors. In order to evaluate discriminative potential of each marker, we have calculated the difference in its methylation frequencies as a simple delta of methylation frequencies in the two groups of samples, as well as individual sensitivity, specificity and area under ROC curve (AUC) for each marker with 100x repeated 5-fold cross-validation (Supplementary Table S1). Methylation frequency differences well correlated with AUC for all the markers with more than 5% methylation frequencies between the two groups of samples (Supplementary Fig. S1).

**Figure 3:**
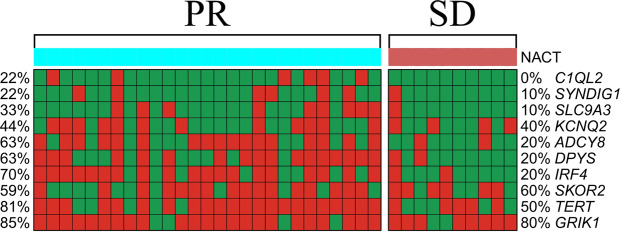
The methylation status and frequencies of 10 differentially methylated genes in 37 luminal B breast cancer samples obtained before surgery. Red color stands for methylated and green stands for non-methylated. Percentages of methylated samples in the groups with PR (partial response) and SD (stable disease) NACT response are shown on the left and on the right respectively.

In order to reduce the number of markers in the final classifier, we have estimated their independence by hierarchical clustering and primary component analyses. Both methods demonstrate that the 10 markers fall into two distinct groups (Supplementary Fig. S2). To select a classifier of the highest possible quality and containing a minimum of markers, we have further calculated ROC characteristics for the combinations of the most independent markers (Supplementary Table S2). Three of the combinations, (1) *IRF4* and *C1QL2*; (2) *IRF4, C1QL2*, and *ADCY8*; (3) *IRF4, C1QL2*, and *DPYS* provided similar ROC characteristics with the AUCs of 0.75, 0.78 and 0.74, respectively (Supplementary Table S2, Supplementary Fig. 3). A classifier based on *IRF4* and *C1QL2* better meets the diagnostic panel simplicity requirements, as it consists of only two markers. Diagnostic accuracy of the panel of these two markers is 0.75, with the sensitivity of 75% and specificity of 75% (Fig. 2b). Logistic regression parameters for this classifier are presented in Table 1.

**Table 1:** Logistic regression parameters for prediction of the Luminal B breast cancer NACT effect based on the methylation status of 2 genes (*IRF4*, *C1QL2*) selected in this study.

Variable	β estimate	Std. error	Odds ratio (CI)
(Intercept)	−0.73	0.60	—
*C1QL2*	1.89	1.60	6.61 (0.28, 152.32)
*IRF4*	1.48	0.91	4.39 (0.73, 26.14)

## Discussion

DNA methylation profiling of the tumor genomes for classifying the subtypes of BC is being considered as an alternative to gene expression assessment^9^. We here report the most informative markers from a genome-wide study of the DNA methylation of BC samples, potentially predictive for tumors response to NACT with FAC, CAX, CMX schemes, or monotherapy with taxotere. All patients included in this study were treated in 2006–2010, when these regimens were practiced, according to the Consensus Conference on Neoadjuvant Chemotherapy in Carcinoma of the Breast, April 26–28, 2003, Philadelphia, Pennsylvania^2^. Although these regimens are no longer a standard for BC NACT, we hypothesize that it is not the regimen but mostly the tumor biological status that predetermines its sensitivity to cytostatic NACT, and this status may be reflected by specific epigenetic signatures that we seek for. At present, this is only a hypothesis that needs to be validated by numerous studies, and our paper is one of the first attempts to approach this validation. It is possible that this hypothesis will not be confirmed by future studies; in such case the relevance of our study lies in presenting a general estimation of how many methylation markers might be required to create a predictor of NACT sensitivity to a given regimen and what sensitivity and specificity one can expect with such approach. Of course, the panel of genes suggested as a result of our study is not intended for immediate use in the diagnostics of breast cancer sensitivity to NACT. It needs more thorough characterization on extended independent cohorts and may undergo changes as information about breast cancer epigenetics accumulates.

The numbers of patients in the cohorts tested in our study is moderate. Overall, we had at our disposal biopsy samples from 62 patients. In contrast to surgery samples, biopsy samples are not usually readily available for research, as far as the bulk of the material is used by pathologists. Thus, the samples of such nature are more difficult to collect, and the quantity of material is far less than that obtained at tumor surgery. From 62 samples, we selected 30 samples of the highest DNA quantity for Cohort 1 to perform genome-wide bisulfite sequencing on, observing the parity between responders and non-responders. Of these, genome-wide bisulfite sequencing results of acceptable quality were obtained for 25 samples. The 5 samples for which genome-wide bisulfite sequencing results of acceptable quality were not obtained, and the rest of the collection were entirely included into Cohort 2. Disparity between responders and non-responders in Cohort 2 might reflect the overall effectiveness of NACT performed at the Oncology Department with the regimens described.

Although with the modest sample size, our study presents the very first experience of identification of differentially methylated genes that may mark the response of breast carcinoma to NACT, by genome-wide bisulfite DNA sequencing. Along with the text we provide a dataset for tumors demonstrating different response to NACT (NCBI GEO database, accession number GSE144221) that will be compatible with other genome-wide methylation sequencing results and will be valuable for the future studies in the field. Additionally, our study demonstrates a means of transfer from genome-wide DNA methylation analysis to a locus-specific assay (multiplex MSRE-PCR) to assess the methylation status of candidate marker genes by a method applicable in clinical diagnostics.

## Material and methods

### Clinical material and treatment

Two cohorts of female patients with luminal B subtype BC of IIa - IIIb (T1-4N0-3M0) clinical stages, who were treated at the General Oncology Department of Tomsk Cancer Research Institute (Tomsk, Russia) in 2006–2010, were included in the present study. Only Luminal B tumors that demonstrated positive ER status, negative HER2 status and Ki67 > 30% by immunohistochemical assays were included in this study. These are classified as “Luminal B-like (HER2-negative)” according to St Gallen Consensus and ESMO Guidelines^1,12^. All tumors were grade 2. This study was conducted in accordance with the Declaration of Helsinki, and was approved by the Institutional Ethics Committee of Tomsk Cancer Research Institute. Written informed consent was obtained from each participant of this study.

Cohort 1 (Table 2) consisted of 25 female patients (the mean age of women was 48.08 ± 8.64). Cohort 2 included 37 patients (Table 2) (the mean age of women was 48.13 ± 7.1). All patients received 2–4 courses of NACT in accordance to the Consensus Conference on Neoadjuvant Chemotherapy in Carcinoma of the Breast, April 26–28, 2003, Philadelphia, Pennsylvania^2^ in following schemes: FAC (5-fluorouracil, adriamycin and cyclophosphamide), CAX (cyclophosphamide, adriamycin and capecitabine), or monotherapy with taxotere, CMX (cyclophosphamide, methotrexate and capecitabine).

**Table 2:** The clinicopathological parameters of breast cancer patients.

Clinicopathological parameters		Cohort 1 N (%)	Cohort 2 N (%)
Age	≤ 48	14 (56)	20 (54.05)
	>48	11 (44)	17 (45.95)
Tumor size	T1	6 (24)	8 (21.62)
	T2	18 (72)	27 (72.97)
	T3	—	1 (2.7)
	T4	1 (4)	1 (2.7)
Lymph node status	N0	8 (32)	14 (37.84)
	N1	14 (56)	20 (54.05)
	N2	1 (4)	1 (2.7)
	N3	2 (8)	2 (5.41)
NACT regimen	CAX	7 (28)	9 (24.32)
	CMX	2 (8)	2 (5.41)
	FAC	13 (52)	16 (43.24)
	Taxotere	3 (12)	8 (21.62)
	FAC (3) + CAX (2)	—	1 (2.7)
NACT response	Partial response	15 (60)	27 (72.97)
	Stable disease	10 (40)	10 (27.03)

Core-biopsy materials obtained from patients prior to NACT were placed in an RNAlater solution (Ambion, USA) and stored at –80 °C (after 24 hours incubation at +4 °C) for further DNA extraction.

Thirty DNA samples of the highest DNA quantity were subjected to genome-wide analysis of DNA methylation using XmaI-RRBS as described below. Of these, genome-wide bisulfite sequencing results of acceptable quality were obtained for 25 samples, which formed cohort 1. Cohort 1 samples were further subjected to MSRE-PCR to assess methylation status of the genes selected based on the genome-wide sequencing, and MSRE-PCR results obtained on cohort 1 were used to develop a limited methylation markers classifier of methylotypes of BC with different response to NACT. Core-biopsy specimens form cohort 2 patients were used for NACT sensitivity classifier validation.

### Evaluation of neoadjuvant chemotherapy

Evaluation of neoadjuvant chemotherapy effect in the group of patients with breast cancer who received treatment was carried out based on the results of clinical examination, breast ultrasound and mammography. Clinical and imaging responses were categorized according to the New Response Evaluation Criteria in Solid Tumours: Revised RECIST Guideline (Version 1.1)^13^. A complete response (CR) was defined as complete disappearance of primary tumor and of lymph node metastasis. A partial response (PR) was determined as a tumor reduction of ≥30%, and stable disease (SD) as a tumor reduction of <30% or a tumor size increase of <20%. Progressive disease (PD) was described as an increase of ≥20% in tumor size^13^.

### DNA isolation and quality control

DNA was obtained using the QIAamp DNA mini Kit (Qiagen, Germany). DNA concentration and purity of the isolation were determined on a NanoDrop-2000 spectrophotometer (Thermo Scientific, USA) (from 50 to 150 ng*/*µL, A260/A280 = 2.10–2.35; A260/A230 = 2.15–2.40). DNA integrity was assessed by capillary electrophoresis using a TapeStation (Agilent Technologies, USA), DNA fragments had a mass of more than 48 kbp.

### Genome-wide DNA methylation analysis

Genome-wide DNA methylation analysis was carried out according to the previously described technology^10^ on an Ion Torrent PGM (Thermo Scientific, USA).

Briefly, genomic DNA extracted from the tumor samples was treated with XmaI restriction endonuclease, and then partially blunted by 5-methyl-cytosines using a Klenow fragment (3′−5′ - exo-). Partially blunt DNA fragments were ligated with 5-methylcytosine-containing adapters. After ligation, the unmethylated adapter chains were nick-translated by adding dATP, dTTP, dGTP, 5-methyl-dCTP and Taq DNA polymerase. DNA fragment libraries prepared as described were size-selected and bisulfite converted. The resulting libraries were purified, their concentration was measured on a fluorometer and high-throughput parallel sequencing was performed on Ion Personal Genome Machine (PGM, Thermo Fisher Scientific, USA).

The sequencing data were processed with standard Ion Torrent Suite™ Software. Alignment was performed against the whole human genome sequence (GRCh37/hg19) using the Bowtie 2 aligner^14^ via the Bismark software^15^.

Using the obtained methylation value (b-value) of each CpG dinucleotide under study we performed cluster analysis of the epigenome-wide data using unsupervised hierarchical clustering. This resulted in the identification of two major clusters of samples, highly methylated and moderately methylated at the CpG islands. These same clusters appeared to be remarkably enriched with the samples of tumors that demonstrated partial response (highly methylated cluster) and stabilization.

### Candidate genes selection

Wilcoxon–Mann–Whitney test was applied to identify CpG dinucleotides that discriminate NACT responding and non- responding tumors; CpG dinucleotides with p-value <0.01 were considered significant. From these, CpG dinucleotides belonging to transcription start sites (TSS) of the human genes were selected and hierarchical clustering was performed with the Manhattan metric and ward.D2 agglomeration method. From the subclusters of the resulting hierarchical tree of CpGs (Fig. 1) we selected as candidate markers CpG dinucleotides that belong to the TSS of the following 15 genes: *CNIH3, PRR5, PTGIS, ATOH1, SNAP25, SLC9A3, C1QL2, DPYS, IRF4, ADCY8, KCNQ2, TERT, SYNDIG1, SKOR2 and GRIK1*, which met the requirements for the implementation of the downstream method of multiplex methylation sensitive restriction enzyme PCR (MSRE-PCR). Requirements for the selection of genome regions for inclusion in the diagnostic panel based on MSRE-PCR were as follows: the presence of three BstHHI recognition sites within a prospective PCR product not exceeding 200 bp, and the length of the DNA fragment flanking the target product that does not contain the BstHHI recognition sites no less than 50 bp.

### DNA hydrolysis with a methylation sensitive restriction enzyme

To analyze DNA methylation at the CpG islands of individual genes, we treated genomic DNA from tissue samples with BstHHI methylation sensitive restriction enzyme (GCG/C recognition site, Sibenzyme, Russia): 20 ng of DNA was mixed with 2 µL of reaction 10x SE Buffer Y (Sibenzyme, Russia) and hydrolyzed with 10U enzyme in a final volume of 20 µL at 50 °C overnight under a layer of mineral oil. Mock digestion was performed in same conditions but with no added enzyme.

### Multilocus MSRE-PCR

Primers design was carried out using the MPprimer 1.4^16^. According to the compatibility matrix in multilocus PCR, the primers were divided into 3 pools (P1, P2, and P3). PCR was performed in parallel with intact DNA samples and samples hydrolyzed with the restriction enzyme BstHHI.

For internal digestive control (DC) of DNA hydrolysis, a fragment of the SNRK gene was used, which was previously shown to be constantly nonmethylated in normal as well as in tumor tissues^17^. As an internal positive control (PC) of amplification, we chose GC-rich regions of the genome that did not contain the recognition sites of the restriction enzyme used, as well as constantly methylated gene fragments.

PCR with primer pools P1 and P2 was performed in 25 µL reaction volume containing 5 ng of genomic DNA, 180 µmol of each dNTP, 68 mmol Tris-HCl pH 8.3 at 25 °C, 16.8 mmol (NH4)2SO4, 0.01% Tween-20, 8% glycerol, 0.1 mg/mL BSA, 3 mmol and 4 mmol MgCl2 for P1 and P2, respectively, 1.5U of Taq DNA polymerase, primers (Table 3), deionized water to final volume. An initial 95 °C denaturation step for 5 min was followed by cycling between 95 °C for 40 s, 70 °C for 40 s and 72 °C for 40 s, 33 times with final elongation for 10 min at 72 °C, under a layer of mineral oil.

**Table 3:** Genomic loci, primer sequences, their final concentrations in PCR reactions and the lengths of the obtained amplicons.

Primers pool	Locus	Primers sequences 5′-3′	Primers concentration (pM)	Product length (bp)
P1	*PC*	F: GTCGGCTCAGGGTCGCTGCTTGG; R: GCTCTAGGCCCGCTTTTCCCCGC	0.3	195
	*DC*	F: CTGGAGGCCCTGCCCTTGCGG; R: CGCCGCACTCGGCCCGCTCC	0.1	95
	*ATOH1*	F: CTCGGTGCAGCTGGACGCTCTGC; R: CCCGTCGCTTCTGTGGGACCGAG	0.03	163
	*C1Ql2*	F: GCATGACTTCCAGGGCGGCGGTG; R: CCTGCCGCATGATCTGCGACCCT	0.04	105
	*CNIH3*	F: TGTCCCGGGGCAGGAGGCAGTTC; R: CGGGCCCTGCAGAGGGTGTCCTA	0.03	110
	*SLC9A3*	F: TTAGCGCGGCCAGAGTCGCTCCC; R: CTGGGCCTGGGGGCTTCGTTGTG	0.03	122
	*SNAP25*	F: TAAGAGTCGCCCCGTGCGGGTGT; R: AGCGAGGGGCGGGAGGAAGTGG	0.04	148
P2	*PC*	F: GTGACGGTGCCACTCACGTCGCC; R: TTCCACTTCGTGCACCGCTCGGC	0.08	197
	*DC*	F: GCCTGGAGGCCCTGCCCTTGC; R: CGCCGCACTCGGCCCGCTCC	0.24	97
	*ADCY8*	F: GCCGGCGTGGGAGAGGACCACTG; R: AACAGCGGAGGAACCGGCTGGCG	0.1	114
	*DPYS*	F: CATCGTTGACCACGCGACCCCCG; R: TCGGTGGGGACCTTGCAGGAGGG	0.32	154
	*IRF4*	F: GCCTCGTGGCTGAAGGGCAGCTC; R: AGCTCACCGCGCTCATGCCGAAC	0.1	145
	*KCNQ2*	F: CCGGCGGCTGCAGAGATGGGAC; R: AGCTGTCTGTCCTGCCCCCTCGG	0.07	105
	*PRR5*	F: CCCTGTTCAGCCTCCGCATTCCCA; R: CCCCAAGGCCTCTGCTGTCCCCT	0.04	167
P3	*PC*	F: GTCGGCTCAGGGTCGCTGCTTGG; R: AGGCCCGCTTTTCCCCGCTTGAG	0.019	190
	*DC*	F: CTCCGCCGCCTCAGTAGCCTCC; R: AGCGCAACTTACTTTCCGCCTGC	0.03	84
	*GRIK1*	F: GCAGTGACGCGGCTCCCCCTTTT; R: CACCAACGCGGGTGTAGCGGGTC	0.015	124
	*PTGIS*	F: GGACTGCCGAAAGCAAGGCAGGG; R: TCTGCGTGGCCCGGGTGGAAGAA	0.04	103
	*SKOR2*	F: CAGAGTCCGCGGGCGGCGTGGAG; R: CCCCCGCAGGTAGTGGCCAACG	0.03	145
	*SYNDIG1*	F: CCGCTAGGGCCTCCCTGGTCTGG; R: GAGCCGCTCCTCTTCGCTTGCCG	0.02	152
	*TERT*	F: GCGGAGCTGGAAGGTGAAGGGGC; R: GGGAAGCGCGGCCCAGACCC	0.027	168

For amplification of P3, GenePak^TM^ PCR Core kits (Isogene, Russia, cat. no. U 1010-05) were used. In a reaction with a lyophilized premix, 10 µL of Diluent PCR, 0.1 µg of DNA, primers (Table 3), and deionized water up to 20 µL were added. PCR was performed under a layer of mineral oil. The reaction mix was heated at 95 °C for 5 min and 33 cycles of PCR were performed with parameters: 95 °C–40 s, 69 °C–40 s, 72 °C–40 s. The final elongation was carried out for 10 min at a temperature of 72 °C.

**Figure 4:**
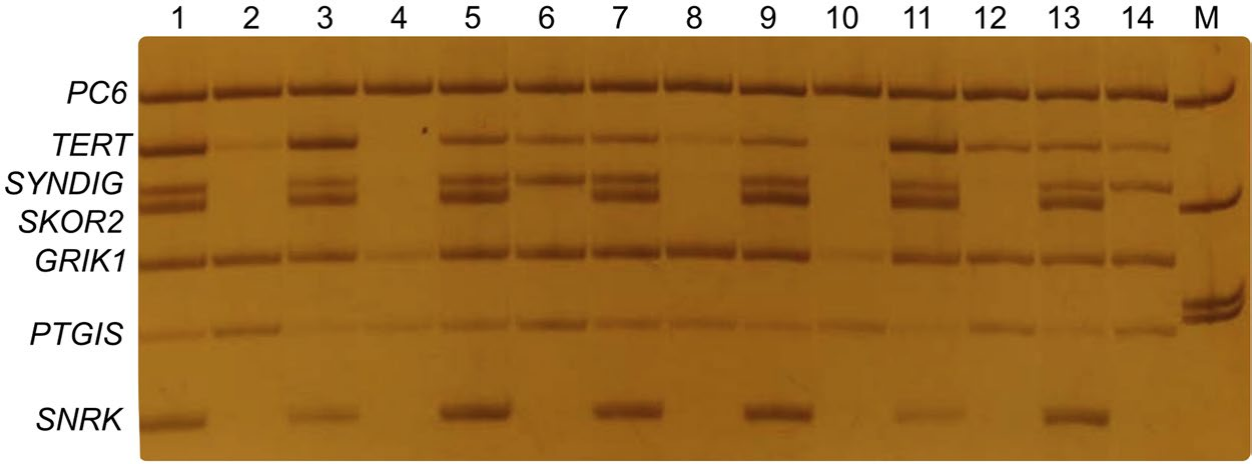
A representative example of multi-locus MSRE-PCR with primers pool P3 for the assessment of DNA methylation at the CpG islands of the genes discriminating good and poor response of tumors to neoadjuvant chemotherapy. Even lanes contain PCR products from tumor DNA samples hydrolyzed with the BstHHI methylation sensitive restriction endonuclease, and odd ones contain PCR products of intact tumor DNA samples (mock digestion without BstHHI). M, DNA molecular weight marker pUC19/MspI.

Amplification products were electrophoresed on an 8% polyacrylamide gel and stained with silver nitrate (Fig. 4).

### Statistical analysis

DNA methylation markers were selected from XmaI-RRBS data using the Wilcoxon–Mann–Whitney test. Assessment of methylation status of the genes under study based on MSRE-PCR products gel electrophoresis results was performed without knowing response to NACT (blinded). The classifiers were built using logistic regression with default cut-off 0.5 to predict NACT response. In view of moderate numbers of patients in the cohorts tested in our study, cross-validation using caret R package^18^ was used to characterize individual markers and their combinations. To assess the independency of markers, PCA (principal component analysis) and hierarchical clustering with the Manhattan distance and ward.D2 agglomeration method were performed. Receiver Operating Characteristics (ROC) curves were plotted using cvAUC R package to assess quality of classification^19^. Most favorable sensitivity and specificity points for classifiers were obtained using Youden’s index. All calculations and plots were performed using statistical programming language R^20^.

## Conclusion

Our study presents the very first experience of identification of differentially methylated genes that may mark the response of breast carcinomas to NACT, by genome-wide bisulfite DNA sequencing. We also demonstrate a means of transfer from genome-wide DNA methylation analysis to a locus-specific assay (multiplex MSRE-PCR) to assess the methylation status of candidate marker genes by a method applicable in clinical diagnostics. Multiplexing of markers in one reaction reduces the requirements for the available material (40 ng), which is critical when conducting research using tumor biopsies. Analytical specificity in our assay is boosted by the simultaneous PCR of the two types of control loci, positive (PC) to verify the PCR amplification, and DNA digestive control (DC) as a reference marker to evaluate the efficiency of the enzyme digestion.

We have developed a classifier based on methylation analysis of 2 markers by multiplex MSRE-PCR, for BC NACT sensitivity prediction. The panel of genes and the test system suggested as a result of our study is not intended for immediate use in the diagnostics of breast cancer sensitivity to NACT. It needs thorough characterization on extended independent cohorts and may undergo changes as information about breast cancer epigenetics accumulates. Limitations to the results presented here relate to the clinical material included in the study. First, only grade 2 breast tumors that demonstrated positive ER and PgR status and Ki67 > 30% by immunohistochemical assays were assessed. Second, all patients included in this study were treated in 2006–2010, with contemporary chemotherapy schemes that are not common at present. Whether differential methylation markers identified in this study are specific for the narrowed group of breast carcinomas that we selected, and/or for the certain chemotherapy schemes, or they reflect the tumor biological status that predetermines its sensitivity to cytostatic NACT more generally, is a matter of further research.

## Supplementary information

Supplementary Information.

## Data Availability

Raw datasets have been submitted to The National Center for Biotechnology Information Gene Expression Omnibus (NCBI GEO) database, accession number GSE144221.
