# Supplementary material for: DNA methylation markers panel can improve prediction of response to neoadjuvant chemotherapy in luminal B breast cancer
Source: Sci Rep. 2020 Jun 8;10:9239. doi: 10.1038/s41598-020-66197-1 (PMC7280523; doi:10.1038/s41598-020-66197-1)
Supplement: Supplementary file 1 — Supplementary Information. [file 41598_2020_66197_MOESM1_ESM.docx]

**DNA methylation markers panel can improve prediction of response to neoadjuvant chemotherapy in luminal B breast cancer**

# Vladimir O. Sigin1,*, Alexey I. Kalinkin1, Ekaterina B. Kuznetsova1,2, Olga A. Simonova1, Galina G. Chesnokova1, Nikolai V. Litviakov3,4, Elena M. Slonimskaya3, Matvey M. Tsyganov3, Marina K. Ibragimova3,4, Ilya V. Volodin1, Ilya I. Vinogradov5,6, Maksim I. Vinogradov6, Igor Y. Vinogradov5, Sergey I. Kutsev1, Vladimir V. Strelnikov1, Dmitry V. Zaletaev1,2, and Alexander S. Tanas1

1Research Centre for Medical Genetics, Moscow, Russian Federation, 115522, Moskvorechie St.1, e-mail: [sigin.vladimir@gmail.com](mailto:sigin.vladimir@gmail.com)

2I.M. Sechenov First Moscow State Medical University, Moscow, Russian Federation, 119991, Trubetskaya St.8, e-mail: [zalnem@mail.ru](mailto:zalnem@mail.ru)

3Tomsk Cancer Research Institute, Tomsk, Russian Federation, 634009, Kooperativniy Lane, 5, e-mail: [nvlitv72@ya.ru](mailto:nvlitv72@ya.ru)

4National Research Tomsk State University, Tomsk, Russian Federation, 634050, Lenin Ave, 36, e-mail:

[nvlitv72@ya.ru](mailto:nvlitv72@ya.ru)

5Pathology and Anatomy Department with Pathology Laboratory, Ryazan Regional Clinical Oncology Dispensary, Sportivnaya St.13, Ryazan, 390011, Russian Federation

6Ryazan State Medical University, Vysokovoltnaya St.9, Ryazan, 390026, Russian Federation

*[sigin.vladimir@gmail.com](mailto:sigin.vladimir@gmail.com)

| **Genes** | **sd_fr,%** | **pr_fr,%** | **fr_delta,%** | **sens,%** | **spec,%** | **auc** | **p.value** | **adj.p.value** |
| --- | --- | --- | --- | --- | --- | --- | --- | --- |
| ***IRF4*** | 20.00 | 70.4 | 50.4 | 80 | 70 | 0.75 | 0.01 | 0.09 |
| ***ADCY8*** | 20.00 | 63.0 | 43.0 | 79 | 62 | 0.71 | 0.03 | 0.10 |
| ***DPYS*** | 20.00 | 63.0 | 43.0 | 80 | 63 | 0.71 | 0.03 | 0.10 |
| ***TERT*** | 50.00 | 81.5 | 31.5 | 50 | 79 | 0.64 | 0.09 | 0.24 |
| ***C1QL2*** | 0.00 | 22.2 | 22.2 | 100 | 19 | 0.60 | 0.16 | 0.32 |
| ***SLC9A3*** | 10.00 | 33.3 | 23.3 | 89 | 31 | 0.60 | 0.23 | 0.38 |
| ***SYNDIG1*** | 10.00 | 22.2 | 12.2 | 87 | 23 | 0.53 | 0.65 | 0.82 |
| ***GRIK1*** | 80.00 | 85.2 | 5.2 | 54 | 60 | 0.48 | 0.65 | 0.82 |
| ***KCNQ2*** | 40.00 | 44.4 | 4.4 | 64 | 55 | 0.57 | 1.00 | 1.00 |
| ***SKOR2*** | 60.00 | 59.3 | 0.7 | 63 | 60 | 0.56 | 1.00 | 1.00 |

**Supplementary Table S1.** individual characteristics of DNA methylation markers evaluated with cross-validation approach after multilocus MSRE-PCR. **sd_fr, pr_fr** – methylation frequencies in stable disease and partial regression groups of tumor samples respectively. **fr_delta -** the difference in methylation frequencies in these groups. Exact Fisher’s test **p.value** and FDR **adj.p.value** are presented in due columns. ROC analysis characteristics for individual markers are shown in **sens** (sensitivity), **spec** (specificity), and **auc** (area under ROC curve) columns. Acceptable p-value was taken as 0.1.

**
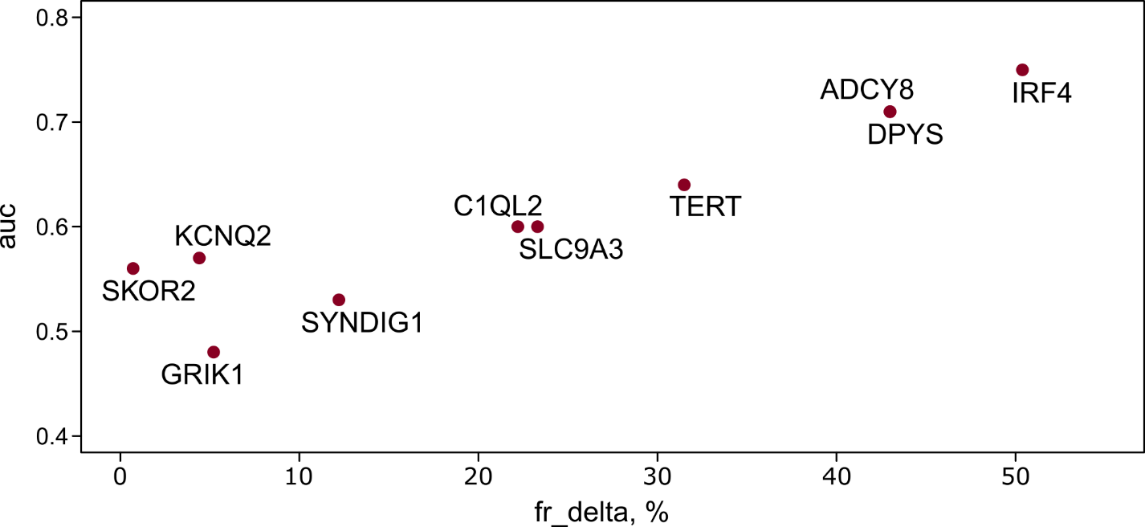
**

**Supplementary Figure S1**. Correlation of AUC for individual markers (Y axis) and the difference in the frequencies of methylation of these markers assessed by MSRE-PCR (X axis).


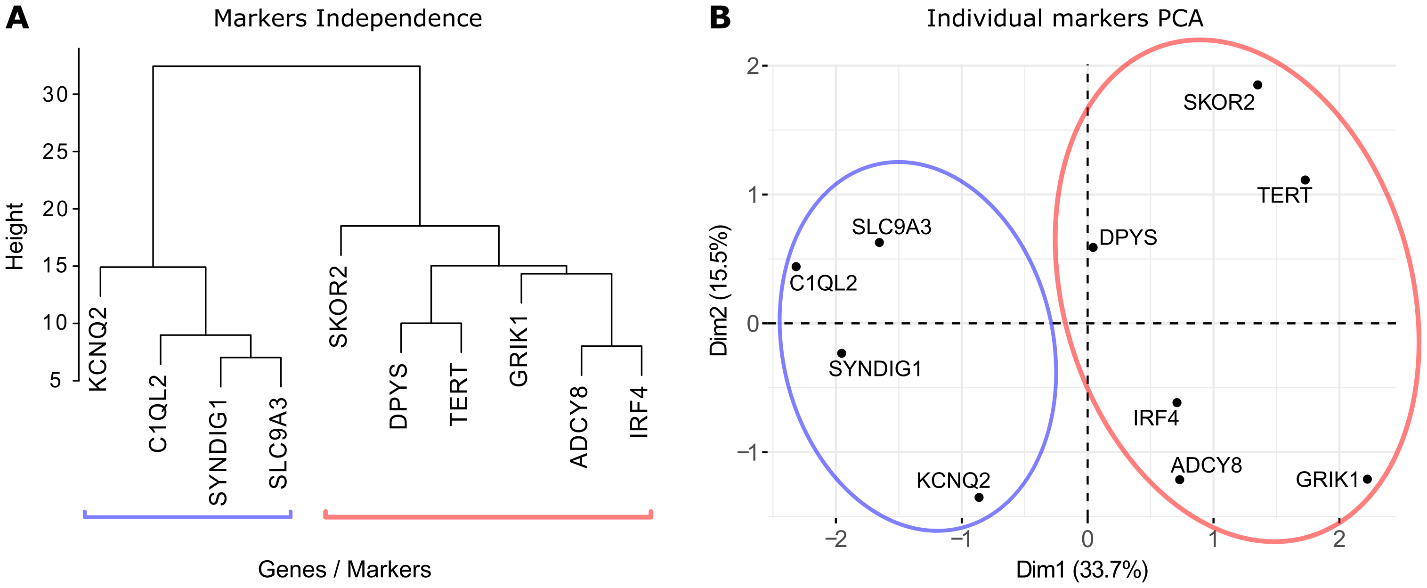


**Supplementary Figure S2.** (**A**) Hierarchical clustering of individual methylation markers assessed by MSRE-PCR. The smaller distance between the markers indicates the more similar behavior of these markers. (**B**) Principle components analysis of individual methylation markers assessed by MSRE-PCR. The plot shows two distinct groups determined according to cluster analysis. The PCA cumulative variance using two principal components, Dim1 (33.7%) and Dim2 (15.5%).

| **Panel** | **auc** | **sens,%** | **spec,%** | **accuracy,%** |
| --- | --- | --- | --- | --- |
| *ADCY8, IRF4* | 0.724 | 55.6 | 85.5 | 63.7 |
| *DPYS, ADCY8* | 0.677 | 70.0 | 67.0 | 69.2 |
| *DPYS, IRF4* | 0.712 | 75.0 | 66.9 | 72.8 |
| ***IRF4, C1QL2*** | **0.750** | 75.0 | 75.4 | 75.1 |
| *DPYS, ADCY8, IRF4* | 0.661 | 68.0 | 65.9 | 67.4 |
| ***IRF4, C1QL2, ADCY8*** | **0.779** | 64.2 | 87.2 | 70.6 |
| ***IRF4, C1QL2, DPYS*** | **0.740** | 75.0 | 70.4 | 73.8 |
| *C1QL2, DPYS, ADCY8* | 0.708 | 72.0 | 68.9 | 71.2 |
| *C1QL2, DPYS, ADCY8, IRF4* | 0.700 | 65.5 | 72.6 | 67.4 |
| *KCNQ2, ADCY8* | 0.626 | 60.0 | 70.9 | 63.0 |
| *KCNQ2, C1QL2, SKOR2, ADCY8* | 0.651 | 56.2 | 72.8 | 60.7 |
| *KCNQ2, C1QL2, SKOR2, GRIK1* | 0.464 | 13.8 | 95.7 | 35.9 |
| *KCNQ2, C1QL2, SKOR2, GRIK1, IRF4* | 0.655 | 60.6 | 71.6 | 63.6 |

**Supplementary Table S2.** Evaluation of ROC (with 100x repeated 5−fold cross-validation) characteristics for candidate combinations of methylation markers.


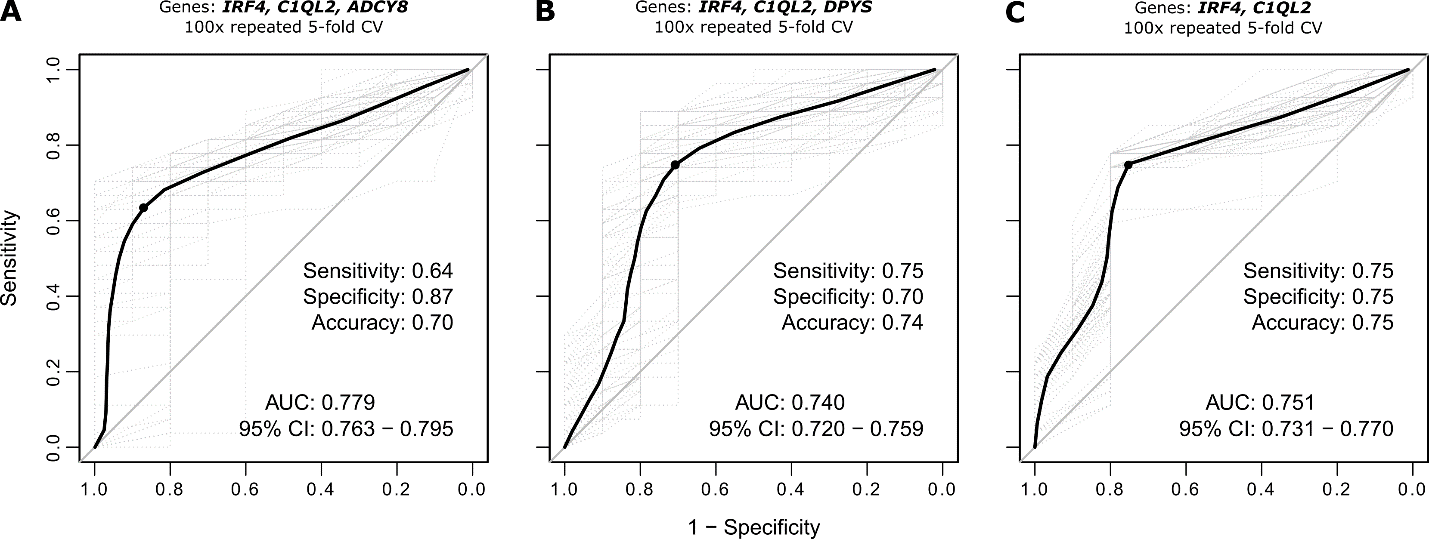
**Supplementary Figure S3.** Evaluation of combinations of methylation markers for the prediction of luminal B breast cancer response to NACT, **(A)** *IRF4, C1QL2*, *ADCY8*; **(B)** *IRF4, C1QL2*, *DPYS*; **(C)** *IRF4*, *C1QL2* by ROC analysis (with 100x repeated 5-fold cross-validation).
